# Adult-Onset Still's Disease With Macrophage Activation Syndrome During Pregnancy: A Unique Cause of Hepatic Dysfunction

**DOI:** 10.14309/crj.0000000000000921

**Published:** 2022-12-26

**Authors:** Thu Anne Mai, Divya Ayyala-Somayajula, Jasleen Singh, David Braxton, Leanna Wise, Liyun Yuan

**Affiliations:** 1 Department of Medicine, University of Southern California, Los Angeles, CA; 2 Division of Gastrointestinal and Liver Disease, Keck School of Medicine, University of Southern California, Los Angeles, CA; 3 Department of Pathology, Hoag Memorial Hospital Presbyterian, Newport Beach, CA; 4 Division of Rheumatology, Keck School of Medicine, University of Southern California, Los Angeles, CA

## CASE REPORT

**Figure 1.**
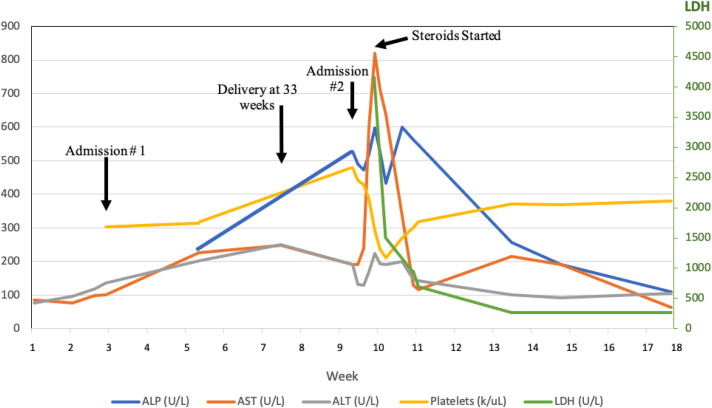
Trajectory of liver tests and function throughout hospital course. ALP, alkaline phosphatase; ALT, alanine transaminase; AST, aspartate aminotransferase; LDH, lactate dehydrogenase.

We present a rare case of adult-onset Still's disease (AoSD) with macrophage activation syndrome (MAS) marked by profound hepatic dysfunction during pregnancy. A 41-year-old woman with a history of hepatic steatosis was initially admitted at 28-week gestation for nausea, vomiting, proteinuria, and mildly elevated transaminases concerning for preeclampsia. Two weeks after admission, she developed daily fevers, an evanescent rash, and rising transaminases (Figure 1) necessitating delivery through caesarean section at 33-week gestation.

**Figure 2.**
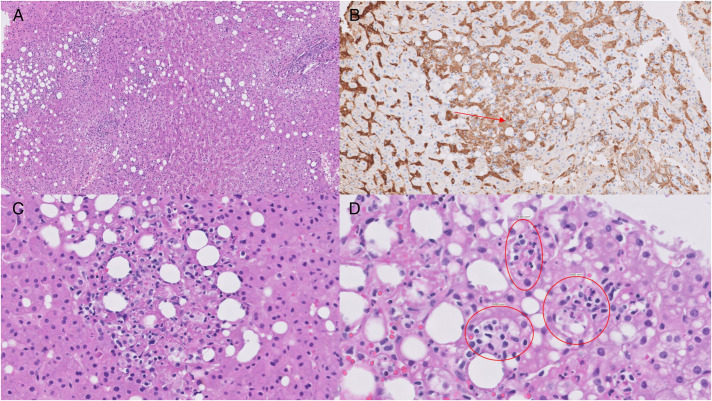
Liver histopathology findings: (A) confluent hepatic necrosis and inflammation (power ×10), (B) positive immunohistochemistry staining for CD163 (arrowed), (C) spot of necrosis, and (D) hemophagocytes (circled).

After delivery, she had persistent fevers and underwent an extensive negative workup for fever of unknown origin. She was treated empirically with antibiotics and underwent a cholecystectomy for possible cholecystitis. Intraoperatively, her liver appeared purplish and friable, so a wedge liver biopsy was performed showing hepatic necrosis and hemophagocytes (Figure 2). Computed tomography abdomen showed hepatomegaly with patent hepatic veins and portal veins. She then developed acute encephalopathy along with worsening coagulopathy and was transferred for expedited liver transplant evaluation.

The patient was diagnosed with AoSD/MAS fulfilling 5 of the Yamaguchi diagnostic criteria for AoSD: 2 major (fevers >1 week and typical rash) and 3 minor (hepatomegaly, abnormal transaminases, and negative rheumatoid factor) criteria. She fulfilled 5 of 8 criteria for MAS under the hemophagocytic lymphohistiocytosis 2004 criteria: fevers, hypertriglyceridemia (465 mg/dL), hypofibrinogenemia (124 mg/dL), hemophagocytosis on liver biopsy, elevated ferritin (>40,000 ng/mL), and elevated soluble interleukin 2 receptor (13,582 pg/mL). She completed a course of dexamethasone and anakinra (interleukin 1 receptor antagonist) daily with resolution of her fevers and normalization of her laboratory results.

In AoSD/MAS, excessive immune activation triggered by proliferating cytotoxic CD8+ T cells and macrophages results in a cytokine storm.^1^ Hepatic manifestations range from mild elevations in transaminases (75%–92%) to hepatomegaly (∼50%) to fulminant liver failure.^2^ The mainstay of treatment for AoSD/MAS is immunosuppression, including anakinra and glucocorticoids but, despite this, mortality rates remain high (8%–22%).^3^ Limited literature exists regarding liver transplant for acute liver failure in AoSD, although outcomes were favorable.^4^ AoSD arising during pregnancy is uncommon and portends significant morbidity for both mother and fetus.^5^ Our case highlights the importance of a timely diagnosis of AoSD/MAS in patients with acute liver injury during pregnancy, given the high mortality and favorable outcomes in response to early treatment.

## DISCLOSURES

Author contributions: TA Mai and D. Ayyala-Somayajula wrote the manuscript. J. Singh, L. Wise, and L. Yuan revised the manuscript. D. Braxton provided the pathology images. L. Yuan revised the manuscript and is the article guarantor. All authors discussed the results and contributed to the final manuscript.

Financial disclosure: L. Yuan has received institutional grant support from Gilead Sciences, Genfit, Intercept, and One-Legacy grant support. The remaining authors disclose no conflicts or grant support.

Previous presentation: This case report was presented as a poster presentation at Digestive Disease Week (DDW); May 24, 2022; San Diego, CA.

Informed patient consent was obtained for this case report.
